# Lack of AcrB Efflux Function Confers Loss of Virulence on *Salmonella enterica* Serovar Typhimurium

**DOI:** 10.1128/mBio.00968-17

**Published:** 2017-07-18

**Authors:** Xuan Wang-Kan, Jessica M. A. Blair, Barbara Chirullo, Jonathan Betts, Roberto M. La Ragione, Alasdair Ivens, Vito Ricci, Timothy J. Opperman, Laura J. V. Piddock

**Affiliations:** a Institute of Microbiology and Infection, College of Medical and Dental Sciences, The University of Birmingham, Birmingham, United Kingdom; b Unit of Prophylaxis and Control of Bacterial Zoonoses, Department of Veterinary Public Health and Food Safety, Istituto Superiore di Sanità, Rome, Italy; c School of Veterinary Medicine, Faculty of Health and Medical Sciences, University of Surrey, Guildford, United Kingdom; d Centre for Immunity, Infection and Evolution, University of Edinburgh, Edinburgh, United Kingdom; e Microbiotix, Inc., Anti-Infectives R&D, Worcester, Massachusetts, USA; University of British Columbia

**Keywords:** AcrB, SPI, *Salmonella*, *Salmonella* pathogenicity island, efflux, motility, transcriptome, virulence

## Abstract

AcrAB-TolC is the paradigm resistance-nodulation-division (RND) multidrug resistance efflux system in Gram-negative bacteria, with AcrB being the pump protein in this complex. We constructed a nonfunctional AcrB mutant by replacing D408, a highly conserved residue essential for proton translocation. Western blotting confirmed that the AcrB D408A mutant had the same native level of expression of AcrB as the parental strain. The mutant had no growth deficiencies in rich or minimal medium. However, compared with wild-type SL1344, the mutant had increased accumulation of Hoechst 33342 dye and decreased efflux of ethidium bromide and was multidrug hypersusceptible. The D408A mutant was attenuated *in vivo* in mouse and *Galleria mellonella* models and showed significantly reduced invasion into intestinal epithelial cells and macrophages *in vitro*. A dose-dependent inhibition of invasion was also observed when two different efflux pump inhibitors were added to the wild-type strain during infection of epithelial cells. RNA sequencing (RNA-seq) revealed downregulation of bacterial factors necessary for infection, including those in the *Salmonella* pathogenicity islands 1, 2, and 4; quorum sensing genes; and *phoPQ*. Several general stress response genes were upregulated, probably due to retention of noxious molecules inside the bacterium. Unlike loss of AcrB protein, loss of efflux function did not induce overexpression of other RND efflux pumps. Our data suggest that gene deletion mutants are unsuitable for studying membrane transporters and, importantly, that inhibitors of AcrB efflux function will not induce expression of other RND pumps.

## IMPORTANCE

Antibiotic resistance is a major public health concern. In Gram-negative bacteria, overexpression of the AcrAB-TolC multidrug efflux system confers resistance to clinically useful drugs. Here, we show that loss of AcrB efflux function causes loss of virulence in *Salmonella enterica* serovar Typhimurium. This is due to the reduction of bacterial factors necessary for infection, which is likely to be caused by the retention of noxious molecules inside the bacterium. We also show that, in contrast to loss of AcrB protein, loss of efflux does not induce overexpression of other efflux pumps from the same family. This indicates that there are differences between loss of efflux protein and loss of efflux that make gene deletion mutants unsuitable for studying the biological function of membrane transporters. Understanding the biological role of AcrB will help to assess the risks of targeting efflux pumps as a strategy to combat antibiotic resistance.

## INTRODUCTION

Antimicrobial resistance (AMR) is a major public health concern (1). Infections caused by AMR microorganisms are more difficult to treat and have been associated with worse disease outcomes (2, 3). Recent studies also suggest that AMR bacteria can be more virulent than their antimicrobial-susceptible counterparts (3, 4). Overexpression of multidrug resistance (MDR) efflux pump genes is a common mechanism of intrinsic and evolved AMR in Gram-negative bacteria (5). We previously reported that a multidrug-resistant (MDR) *Salmonella enterica* serovar Typhimurium (*S*. Typhimurium) strain with a mutated AcrB MDR efflux pump was responsible for failure of treatment in a 52-year-old patient (6, 7). MDR efflux pumps are integral inner membrane transporters that confer resistance to multiple antibiotics and other chemicals (5). AcrB belongs to the resistance-nodulation-division (RND) family, members of which are the main MDR transporters in Gram-negative bacteria. RND pumps function as drug/proton antiporters. They associate with an outer membrane protein and a periplasmic adapter protein to form a tripartite MDR efflux system (8). The best-studied RND efflux system is AcrAB-TolC, formed by the AcrB RND efflux pump, the TolC outer membrane protein, and the AcrA periplasmic adapter protein. This system is the most abundant RND efflux complex in the *Enterobacteriaceae* family (9).

AcrAB-TolC of *Escherichia coli* has been the main focus of study for structural studies, giving important information about the stoichiometry of the system, assembly and disassembly, substrate (drug) binding pockets and molecular interactions, and the proton translocation pathway (8–12). However, to investigate the relationship between MDR efflux and virulence, tractable infection models are required. *Salmonella enterica* is an invasive human pathogen that has a high genetic similarity to *E. coli* and for which there are multiple infection models. The AcrB pump has 97% amino acid similarity between the two species; thus, *Salmonella* provides an ideal model to study the role of the pump in the overall biology of the bacterium. Furthermore, the enterobacterial AcrB is highly conserved with those of other bacterial species, such as *Pseudomonas* spp. and *Acinetobacter* spp.; thus, the results obtained from *Salmonella* can be translated to other bacteria. Previous studies have shown that AcrB-deficient mutants of *S*. Typhimurium exhibit impaired invasion of human intestinal epithelial cells and murine macrophages. The *acrB*::*aph* mutant did not induce membrane ruffles, as shown by electron microscopy, and could not colonize chick intestinal tracts (13). Further studies of the transcriptome indicated a large number of gene expression changes in the *acrB*::*aph* mutant, including the downregulation of *Salmonella* pathogenicity island (SPI) genes that are essential for invasion (14).

RND efflux systems are also required for virulence in many other species. In *Enterobacter cloacae* and *Klebsiella pneumoniae*, deletion of *acrAB* resulted in impaired ability of the mutants to cause systemic infection (15) and pneumonia in mice (16), respectively. Likewise, deletion of the *acrAB-oprM* system in *Moraxella catarrhalis* decreased invasion of the mutant into pharyngeal epithelial cells (17). In *Vibrio cholerae*, RND efflux systems are essential for the synthesis of toxins and colonization of the small intestine in mice (18). Similarly, CmeABC from *Campylobacter jejuni* is needed for gut colonization of 1-year-old chicks and has been recently associated with expression of a type 6 secretion system (T6SS) (19). In *Pseudomonas aeruginosa*, RND efflux pumps play a dual role in virulence as they export quorum sensing (QS) autoinducers (AIs). Thus, deletion of RND pumps causes loss of virulence due to the absence of AIs, but overexpression of them also causes loss of virulence, as AIs will not accumulate inside the cells to levels required to activate QS-controlled virulence genes (19). Furthermore, the MtrCDE RND efflux system in *Neisseria gonorrhoeae* is required for infection of the genitourinary system in female mice (20). The virulence-related function of RND efflux pumps has also been described in the phytopathogen *Erwinia amylovora*, which is less virulent when *acrAB* is deleted (21).

A criticism that has been raised against all the previous studies is that RND pump gene deletion mutants were used. RND efflux pumps are transmembrane proteins that span the inner membrane of Gram-negative bacteria up to 12 times. Therefore, it is not clear whether the absence of a large integral membrane protein or the loss of efflux activity is responsible for the published phenotypes.

In the present study, a mutant incapable of efflux activity via AcrB was constructed by introducing a D408A substitution into the protein via a single nucleotide change in the chromosomal *acrB* gene of *S*. Typhimurium SL1344. The D408A substitution disrupted proton translocation in the pump and was confirmed to abolish efflux. The effect of loss of efflux on virulence in *S*. Typhimurium SL1344 was studied in mouse, *Galleria mellonella*, and tissue culture infection models, and the global effect of loss of efflux in the biology of *Salmonella* was determined by RNA sequencing (RNA-seq). Our data show that the loss of virulence in AcrB-defective mutants is due to loss of efflux pump activity and is not a secondary effect of the absence of an important integral protein. However, we also show that there are differences in transcription between an Δ*acrB* mutant and the loss-of-function mutant. This suggests that gene deletion/inactivation mutants are unsuitable for studying the physiological role of efflux pumps.

## RESULTS

### The AcrB D408A substitution does not affect bacterial growth and confers susceptibility to AcrB substrates by abolishing efflux.

The presence of a T→G substitution at position 530662 in the chromosome of the D408A mutant was confirmed by whole-genome sequencing (WGS). This translates into the D408A substitution in AcrB (see Fig. S1A in the supplemental material). The expression of AcrB in the D408A mutant was confirmed by Western blotting to be similar to that of the parental strain (100% versus 102%, respectively) (Fig. S1B).

10.1128/mBio.00968-17.2 FIG S1  SNP and Western blot analysis of AcrB of the *S*. Typhimurium SL1344 wild type and *acrB* mutants. (A) Identification of the SNP corresponding to the AcrB D408A substitution. An asterisk at the end of the “Mutation” column denotes the SNPs present in wild-type SL1344. Marked in yellow is the *acrB* SNP corresponding to the AcrB D408A mutation. This SNP is shown in the Artemis plot where each one of the blue and green lines represents a mapped read and the vertical red lines indicate SNPs; only red lines that go across all the reads are real SNPs. (B) Bands corresponding to AcrB and the β subunit of the RNA polymerase are shown in the figure. The RNA polymerase Western blot confirms that there is an equal amount of protein in each well. Band intensity was determined using Genesys; this information was used to assign a relative concentration of AcrB to each band, expressed as percentage of wild-type value. An Δ*acrB* mutant, in which the gene was deleted from the chromosome, was used as a negative control. The figure is representative of three independent experiments. Download FIG S1, TIF file, 3.4 MB. Copyright © 2017 Wang-Kan et al. 2017 Wang-Kan et al. This content is distributed under the terms of the Creative Commons Attribution 4.0 International license.

The growth of the AcrB D408A mutant in rich and minimal medium was used to determine the effect of the amino acid substitution upon the fitness of the mutant. The AcrB D408A mutant and the Δ*acrB* mutant had a similar generation time as the parental strain, SL1344 (Fig. S2). These data indicate that the mutation did not confer a detectable growth defect *in vitro*.

10.1128/mBio.00968-17.3 FIG S2  Growth of *S*. Typhimurium SL1344 and its *acrB* mutants. (A) Growth curve and generation times of *S*. Typhimurium strains in LB broth. (B) Growth curve and generation times of *S*. Typhimurium strains in MOPS minimal medium. The growth curves represent the mean from 3 experiments. Download FIG S2, TIF file, 0.5 MB. Copyright © 2017 Wang-Kan et al. 2017 Wang-Kan et al. This content is distributed under the terms of the Creative Commons Attribution 4.0 International license.

We next determined the susceptibility of the mutant to AcrB substrates. As shown previously, the SL1344 Δ*acrB* mutant was multidrug and dye hypersusceptible. Likewise, the D408A mutant was hypersusceptible to all the substrates tested, with at least a 2-fold dilution decrease in the MICs of all compounds compared to those for the parental strain (Table 1).

**TABLE 1:** MICs of AcrB substrates for SL1344 and *acrB* mutants

Strain	MIC (µg/ml) of substrate^a^:													
	ACR	ETBR	CIP	NAL	CHL	TET	NOV	FUS	ATM	CAZ	CTX	ERY	OX	MIN
SL1344	512	>2,048	0.030	4	8	2	512	2,048	0.50	1	0.500	256	512	2
Δ*acrB* mutant	64	32	0.008	1	2	0.5	8	32	0.12	0.25	<0.008	8	16	0.25
AcrB D408A	32	32	0.008	1	2	0.5	8	16	0.12	0.25	<0.008	8	16	0.25

a Abbreviations: ACR, acriflavine; ETBR, ethidium bromide; CIP, ciprofloxacin; NAL, nalidixic acid; CHL, chloramphenicol; TET, tetracycline; NOV, novobiocin; FUS, fusidic acid; ATM, aztreonam; CAZ, ceftazidime; CTX, cefoxitin; ERY, erythromycin; OX, oxacillin; MIN, minocycline. Mode values from four experiments are shown.

**FIG 1:**
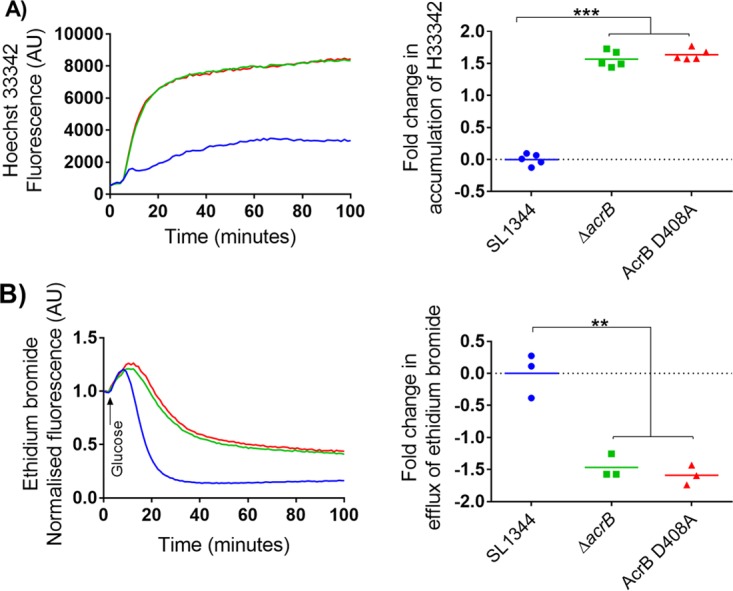
Accumulation of H33342 and efflux of ethidium bromide in *S*. Typhimurium SL1344 and *acrB* mutants. (A) Accumulation kinetics and final fold change in accumulation of H33342. (B) Efflux kinetics and final fold change in efflux of ethidium bromide (ETBR). Double and triple asterisks denote a significant difference of *P* < 0.005 and *P* < 0.0001, respectively. Data were analyzed with Student’s *t* test with Welch’s correction. Blue, green, and red curves in left panels correspond to SL1344, Δ*acrB*, and AcrB D408A strains, respectively.

To confirm that the MIC results were a consequence of loss of efflux, the efflux activity of all strains was measured by determining the intracellular accumulation of Hoechst 33342 (H33342) and efflux of ethidium bromide (22). Both dyes are MDR efflux pump substrates that fluoresce when bound to double-stranded nucleic acids (23). A time-dependent increase in fluorescence was observed as the H33342 dye accumulated inside the bacterial cells until the system came to an equilibrium/steady state. The *acrB* mutants reached equilibrium faster than SL1344 and had a significantly higher accumulation rate of approximately 2-fold more dye than the parental strain (Fig. 1A). To measure efflux of ethidium bromide, bacteria were preloaded with the dye and subsequently energized with glucose to initiate efflux, which resulted in a decrease in fluorescence over time. The *acrB* mutants reached a steady state faster than SL1344, and efflux was approximately 2-fold lower than in the parental strain (Fig. 1B).

### The AcrB D408A mutant has decreased virulence.

As the mutant did not have growth defects *in vitro* and had a loss of efflux function, we determined the effect of the D408A mutation on virulence. This was assessed in three infection models: a BALB/c mouse model, a *Galleria mellonella* model, and the gentamicin protection tissue culture model.

**FIG 2:**
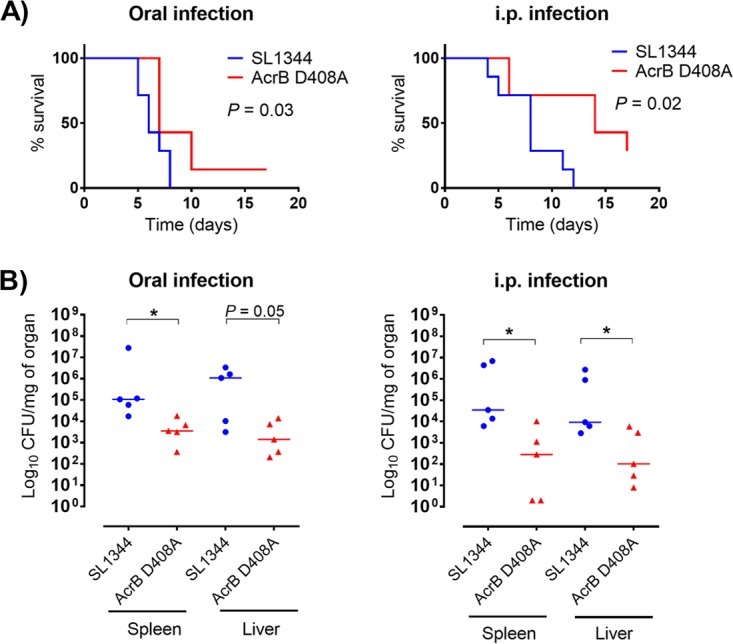
Survival curves and CFU per organ of BALB/c mice infected via oral inoculation or intraperitoneal inoculation. (A) Kaplan-Meier survival curves of mice infected with SL1344 or its AcrB D408A mutant for 17 days. *P* values were determined by the log rank (Mantel-Cox) test. A *P* value of <0.05 denotes a significant difference. (B) CFU per organ of bacteria recovered from spleen and liver at 5 days postinfection. The median is plotted. An asterisk denotes a significant difference of *P* < 0.05 between two groups, compared by the Mann-Whitney U test.

To assess the overall virulence of the mutant compared with the wild type, survival curves and systemic dissemination by oral or intraperitoneal (i.p.) infection were determined. Oral infection was used to determine dissemination through the natural infection route, whereas i.p. infection was used to assess the ability of the mutant to colonize and persist in organs via direct invasion of the mononuclear phagocyte system (MPS) without going through the enteric barrier. Mice infected with the D408A mutant had a significantly better outcome than those infected with the wild type as shown by the survival curves (Fig. 2A). In the oral infection model, the CFU of mutant per milligram of organ was significantly lower in the spleen. The same trend was observed in the liver; however, there was no significant difference between mutant and wild type (*P* = 0.05) (Fig. 2B). Mice infected via the i.p. route had a significantly lower bacterial load in spleen and liver than those infected with the wild type (Fig. 2B).

**FIG 3:**
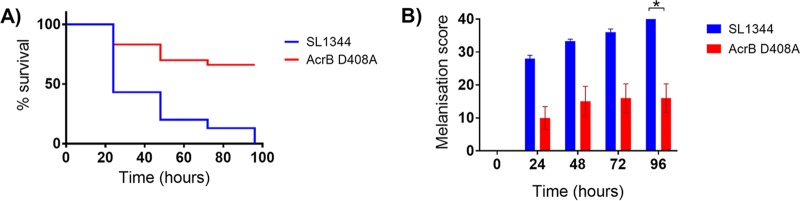
Survival curves and morbidity (melanization) in *Galleria mellonella*. Kaplan-Meier survival curves (A) and melanization (B) of *G. mellonella* infected with SL1344 or the AcrB D408A mutant are shown. An asterisk denotes a significant difference of *P* < 0.05 between two groups. Analyses were done by Student’s *t* test.

Wild-type SL1344 displayed significantly (*P* < 0.05) greater virulence in *G. mellonella* than the AcrB D408A mutant, with 0% larval survival at 96 h compared to 66% ± 14% survival, respectively (Fig. 3A). Significant differences (*P* < 0.05) in melanization between wild type and mutant were also observed over 96 h (Fig. 3B).

**FIG 4:**
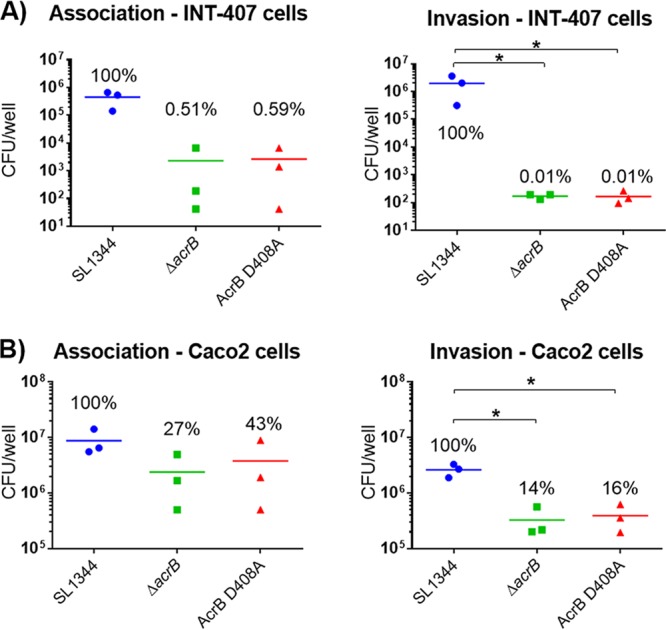
Infection assays with SL1344 and its *acrB* mutants in INT-407 (A) and Caco2 (B) intestinal epithelial cells. Association (2 h postinfection) and invasion (4 h postinfection) of the Δ*acrB* and the AcrB D408A mutants into INT-407 and Caco2 cells were compared to those of the parental wild-type strain. An asterisk denotes a significant difference of *P* < 0.05 between two groups. Analyses were done by Student’s *t* test with Welch’s correction.

**FIG 5:**
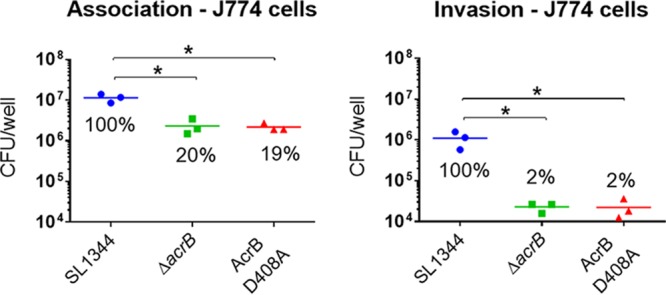
Infection assays with SL1344 and its *acrB* mutants in J774 murine macrophages. Association (2 h postinfection) and uptake (4 h postinfection) of Δ*acrB* and AcrB D408A mutants into J774 murine macrophages were compared to those of the parental wild-type strain. An asterisk denotes a significant difference of *P* < 0.05 between two groups, compared by Student’s *t* test with Welch’s correction.

The mutant was also attenuated in tissue culture cells. Association and invasion of bacteria into intestinal epithelial cells and macrophages were measured by gentamicin protection assays at 2 and 4 h postinfection. Nonpolarized INT-407 and polarized Caco2 intestinal epithelial cells were used in the infection assays. The invasions of all cell lines by the D408A and the Δ*acrB* mutants were similar. The *acrB* mutants invaded the intestinal epithelial cells significantly less than the parental strain (Fig. 4). The same was found with infection of J774 murine macrophages (Fig. 5).

### Efflux pump inhibitors decrease association and invasion of *S*. Typhimurium SL1344 in a dose-dependent manner.

To further determine whether the observed decrease in virulence was due to loss of efflux function, tissue culture assays on INT-407 cells were carried out with the parental SL1344 strain and various concentrations of efflux pump inhibitors: phenylalanine-arginine β-naphthylamide (PAβN) and MBX3132. PAβN is a peptidomimetic compound and was the first efflux pump inhibitor (EPI) described, and recent studies have shown that it binds to the inner binding pocket of AcrB (12). MBX3132 is a new EPI; it is a pyranopyridine derivative that also binds to the inner binding pocket of AcrB but with much higher affinity than PAβN (24). The ranges of concentrations tested were chosen from the literature based on their inhibitory effect on efflux of *P. aeruginosa* and *E. coli* (24, 25). The efflux-inhibitory effect of these EPIs was tested on SL1344 using the H33342 and ethidium bromide accumulation and efflux assays. The EPIs showed a dose-dependent inhibitory effect upon accumulation and efflux of the dyes (Fig. S3).

10.1128/mBio.00968-17.4 FIG S3  H33342 accumulation and ethidium bromide efflux assays with PAβN (A) and MBX3132 (B). (A) Inhibitory effect of PAβN on efflux of ethidium bromide by SL1344 wild-type strain. (B) Inhibitory effect of MBX3132 on efflux of SL1344 by increasing intracellular accumulation of H33342 and decreasing efflux of ethidium bromide. An asterisk denotes a significant difference of *P* < 0.05 compared with 0 μg/ml. Download FIG S3, TIF file, 0.4 MB. Copyright © 2017 Wang-Kan et al. 2017 Wang-Kan et al. This content is distributed under the terms of the Creative Commons Attribution 4.0 International license.

**FIG 6:**
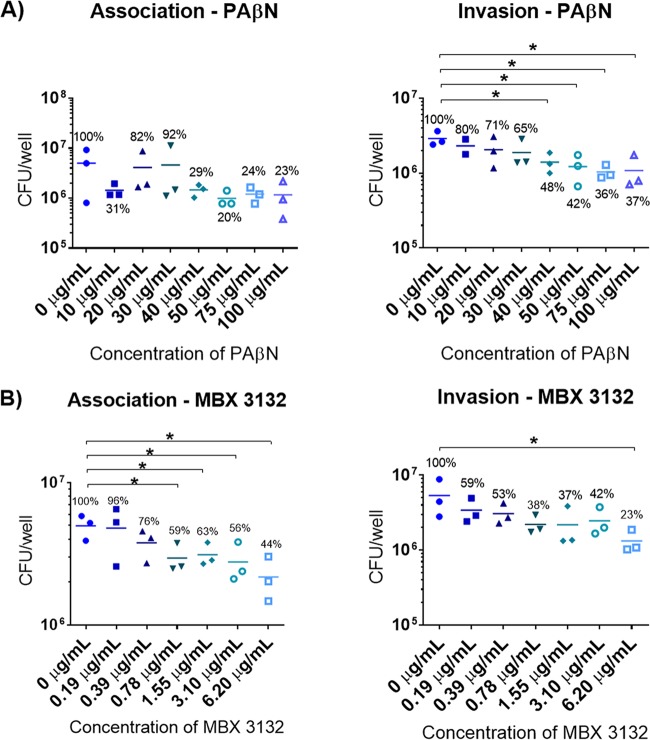
Effect of PAβN (A) and MBX3132 (B) on association and invasion of *S*. Typhimurium SL1344 into INT-407 cells. PAβN and MBX3132 were dissolved in sterile distilled water and dimethyl sulfoxide, respectively. Final concentrations were adjusted in inoculation medium. The 0-μg/ml concentration of MBX3132 contains 1% dimethyl sulfoxide. Association and invasion were measured at 2 h and 4 h postinfection, respectively. Percentages relative to 0 μg/ml are indicated in the figure. An asterisk denotes a significant difference of *P* < 0.05 between two groups, compared by Student’s *t* test with Welch’s correction.

The effect of the EPIs on association and invasion of intestinal epithelial cells was determined. Concentrations of PAβN ranging from 10 to 100 μg/ml (34.4 to 344.4 μM) were used for the infection assays. These had no apparent effect on the viability of eukaryotic cells or bacteria (Fig. S4A) and no significant effect on association of SL1344 with the cells (Fig. 6A). However, PAβN significantly affected invasion of SL1344 at concentrations of ≥30 μg/ml (103.3 μM), in a dose-dependent manner (Fig. 6A). MBX3132 at concentrations of 0.19 to 6.2 μg/ml (0.39 to 12.5 μM) did not affect the viability of eukaryotic cells or bacteria (Fig. S4B). Over this concentration range, MBX3132 decreased association and invasion in a dose-dependent manner. Association of SL1344 was significantly decreased by MBX3132 at concentrations as low as 0.78 μg/ml (1.56 μM); however, invasion was not significantly decreased until 6.20 μg/ml (12.5 μM) (Fig. 6B). These results confirmed that inhibition of efflux activity decreased the ability of SL1344 to infect human epithelial cells.

10.1128/mBio.00968-17.5 FIG S4  Growth kinetics of SL1344 and viability of INT-407 cells with various concentrations of PAβN (A) and MBX3132 (B). Concentrations of PAβN between 10 and 200 µg/ml were tested. MBX3132 was tested in concentrations in 2-fold increases starting from 0.19 µg/ml (0.39 µM). The 0-μg/ml concentration of MBX3132 contains 1% dimethyl sulfoxide, which was the solvent used to dissolve the compound. Download FIG S4, TIF file, 0.4 MB. Copyright © 2017 Wang-Kan et al. 2017 Wang-Kan et al. This content is distributed under the terms of the Creative Commons Attribution 4.0 International license.

### RNA-seq reveals downregulation of SPI genes and upregulation of stress response and flagellar motility genes.

To understand the physiological changes underpinning the loss of virulence in the D408A mutant, changes in gene expression in the mutant compared with SL1344 were assessed by sequencing RNA isolated from each strain. This experiment was carried out in minimal medium as it simulates the nutrient availability conditions inside host cells (26). Quantitative reverse transcriptase PCR (qRT-PCR) confirmed the RNA-seq data (Fig. S5). Compared to the transcriptome of SL1344, 14% of the genome of the mutant was differentially transcribed; the majority of these genes were involved in metabolic processes (Fig. S6). Transcription of some of the major outer membrane porins, such as those encoded by *ompA* and *ompF*, was downregulated. Interestingly, the transcription levels of other RND efflux pump genes were not significantly changed.

10.1128/mBio.00968-17.6 FIG S5  Validation of RNA-seq results by qRT-PCR. Transcription of selected genes in the D408A mutant was compared to that in the wild-type strain by RNA-seq (*x* axis) and qRT-PCR (*y* axis). All values are presented as log_2_ fold change. Download FIG S5, TIF file, 0.3 MB. Copyright © 2017 Wang-Kan et al. 2017 Wang-Kan et al. This content is distributed under the terms of the Creative Commons Attribution 4.0 International license.

10.1128/mBio.00968-17.7 FIG S6  Classification of differentially transcribed genes into COG groups and classes. Each COG group is comprised of different classes. The percentage of differentially expressed genes in each class was calculated by dividing the number of genes changed per class by the total number of genes in the class. Download FIG S6, TIF file, 2.9 MB. Copyright © 2017 Wang-Kan et al. 2017 Wang-Kan et al. This content is distributed under the terms of the Creative Commons Attribution 4.0 International license.

**FIG 7:**
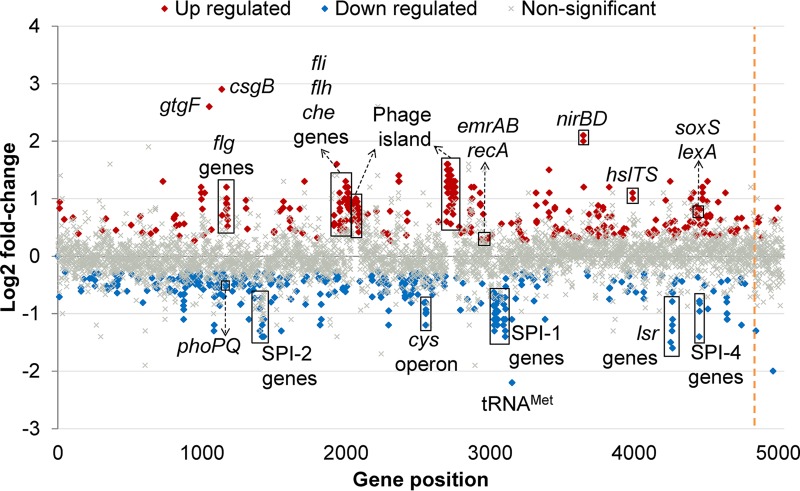
Transcriptional landscape of the SL1344 AcrB D408A mutant. The transcriptome of the mutant was compared to that of parental SL1344. Significantly upregulated genes in the mutant are marked in red, and significantly downregulated genes are marked in blue. Gray crosses denote genes with no significant changes between the two strains. The dashed orange line indicates the end of the chromosomal genes and the start of the plasmid genes.

Levels of expression of several tRNAs were significantly different in the mutant from SL1344. The most downregulated gene was a methionine tRNA (tRNA^Met^) (log fold change of −2.2 compared to wild type). There was also downregulation of the *lsr* quorum sensing transporter operon and the *phoPQ* two-component system. Upregulation of two entire phage islands and several phage-related genes was also observed. The *gtgF* phage gene and *csgB* curli gene were the most upregulated genes (log fold changes of 2.6 and 2.9, respectively) (Fig. 7).

Cluster of orthologous gene (COG) analysis revealed that most of the genes involved in carbohydrate, amino acid, lipid, and nucleotide transport and metabolism were downregulated (Fig. S7). The majority of the genes in the “intracellular trafficking, secretion and vesicular transport” class were also downregulated. This COG class includes SPI genes, which we previously reported as being downregulated in an *acrB*::*aph* mutant (14). All the differentially transcribed motility-related genes were upregulated, including the *fli* and *flh* operons and several *che* genes. In fact, cell motility was the only COG class for which all the genes were upregulated (Fig. S7).

10.1128/mBio.00968-17.8 FIG S7  Percentage of differentially transcribed genes in each COG class. The classes are presented on the left side of the chart; the total number of genes in this class is indicated in brackets. Percentages of up- and downregulated genes in each class are denoted with red and blue bars, respectively. Download FIG S7, TIF file, 1.9 MB. Copyright © 2017 Wang-Kan et al. 2017 Wang-Kan et al. This content is distributed under the terms of the Creative Commons Attribution 4.0 International license.

Interestingly, the “posttranslational modification, protein turnover, chaperones” and “replication, recombination and repair” classes were mainly upregulated. These classes comprise genes encoding heat shock proteins (HSPs) and DNA polymerases and topoisomerases. Surprisingly, the SOS system regulators *lexA* and *recA* were also upregulated, but none of the SOS system effectors were significantly transcribed.

### The AcrB D408A substitution increases swimming motility in minimal medium.

Our RNA-seq data suggested that the D408A mutant has increased motility. To test this hypothesis, motility assays in minimal medium supplemented with 0.3% agar were carried out. Swimming of an Δ*acrB* mutant and the D408A mutant was compared against the parental SL1344 strain. The Δ*acrB* and the D408A mutants swam significantly more than the parental strain (Fig. S8).

10.1128/mBio.00968-17.9 FIG S8  Swimming motility assay in 0.3% agar. (A) Motility assay results from SL1344 and its *acrB* mutants. (B) The area of motility was measured by ImageJ and plotted. Two asterisks denote a significant difference of *P* < 0.01 between two groups, compared by Student’s *t* test. Download FIG S8, TIF file, 0.3 MB. Copyright © 2017 Wang-Kan et al. 2017 Wang-Kan et al. This content is distributed under the terms of the Creative Commons Attribution 4.0 International license.

### Homologous RND efflux pumps are not upregulated in the D408A mutant.

Previous studies have shown that *acrD* and *acrF* genes encoding homologous RND pumps in enterobacteria were significantly upregulated in an Δ*acrB* mutant, by qRT-PCR. These genes were not significantly transcribed in the D408A mutant; therefore, qRT-PCR was carried out to confirm their transcript levels. Either *acrD* or *acrF* was significantly upregulated in the D408A mutant (Table 2).

**TABLE 2:** Transcription of *acrBDF* pumps and *ramA* in the D408A mutant compared with Δ*acrB* mutant

Strain	Fold change in transcription of gene^d^:			
	*acrB^a^*	*acrD^a^*	*acrF^a^*	*ramA^b^*
Wild type	1.0	1.0	1.0	1.0
Δ*acrB* mutant^a^	—^c^	13.4^c^*	16.0^c^*	5.2*
AcrB D408A	1.3	1.2	1.0	1.0

a Data from qRT-PCR.

b Data from *gfp* promoter fusion reporter.

c Data taken from reference 54.

d An asterisk denotes significantly increased transcription (*P* < 0.05).

## DISCUSSION

We focused on investigating the biological function of the AcrB MDR efflux pump and the impact of loss of efflux function on the virulence of *Salmonella* Typhimurium. Previous studies have shown that *acrB* insertional inactivated/deleted mutants of *Salmonella* are less virulent than the wild-type strain (13, 14, 27). However, whether this phenotype was due to loss of a large integral membrane protein or to loss of efflux function has remained an ongoing debate. Several other authors have also observed loss of virulence in RND efflux pump deletion mutants across a range of bacterial species, but none have shown evidence of the phenotype being a consequence of loss of efflux function (13, 15–18, 20, 28, 29). Thus, we constructed an AcrB D408A loss-of-efflux-function mutant with the gene *in situ* in the chromosome and which expressed AcrB at native levels. Five residues are essential for proton translocation by the AcrB transporter, including D408 (30). D408 is crucial for proton translocation as intrinsic resistance to AcrB substrates is lost when this residue is replaced by alanine or asparagine but not by glutamine (31). The D408A substitution locks AcrB in one conformation, rendering it incapable of efflux (30).

The *Salmonella* AcrB D408A mutant was less virulent in a mouse model, where mice infected with the mutant had a better disease outcome than those infected with the wild type (which correlated with data for a deletion mutant [32]) and had lower bacterial loads in spleen and liver. The loss of virulence was also observed in a *G. mellonella* model and in cultured intestinal epithelial cells and macrophages, a finding which has been previously reported for the deletion mutant (13). It was also shown that SL1344 lost its ability to associate with and invade intestinal epithelial cells as the concentration of each of two EPIs increased, further indicating that loss of virulence was due to loss of AcrB efflux function.

To investigate the role that AcrB plays in virulence, we compared the transcriptome of the mutant with that of the wild-type strain. Results identified two possible mechanisms (not mutually exclusive): (i) decreased production of virulence effectors and (ii) increased stress responses.

SPI-1, -2, and -4 (type 3 secretion system virulence effectors and apparatus genes) were significantly downregulated in the D408A mutant. These are required by *S*. Typhimurium to invade and survive in the host cells, which will result in a systemic infection in mice. Infection in mice starts when *S*. Typhimurium crosses the enteric barrier by invading M cells and intestinal epithelial cells, followed by invasion of macrophages and dissemination via the MPS. *S*. Typhimurium requires SPI-1 and SPI-4 to invade polarized epithelial cells and only SPI-1 to invade nonpolarized cells (33). Therefore, the downregulation of SPI-1 and SPI-4 in the D408A mutant could account for the low levels of invasion into intestinal epithelial cells. In contrast, invasion of macrophages occurs primarily by phagocytosis (34). Survival inside macrophages depends largely on expression of SPI-2 effectors, which are regulated by the PhoPQ two-component system (35). The downregulation of *phoPQ* and SPI-2 genes leads to decreased survival inside macrophages and neutrophils (36). In the D408A mutant, *phoPQ* and SPI-2 were significantly downregulated, which is likely to be the cause of the decreased virulence of the mutant in macrophages. The accrued downregulation of all these virulence factors is likely to be the cause of attenuation of virulence observed in the mouse model.

Alongside SPI genes and *phoPQ*, we also observed decreased transcription of quorum sensing (QS) transporter genes *lsrACDBK* and the operon repressor *lsrR* in the D408A mutant. QS is used by multiple bacterial species to coordinate the expression of virulence factors. The relationship between QS and virulence in *Salmonella* has not been completely elucidated (37, 38); however, it has been suggested that *lsrR* controls expression of SPI-related genes (38). Therefore, it is possible that the downregulation of SPI genes is related to the downregulation of *lsr* genes.

All flagellar synthesis genes in the D408A mutant were significantly upregulated. Flagella play a role in motility and early stages of invasion (39–41). The upregulation of flagellar genes in the D408A mutant translated into a hypermotile phenotype in the D408A mutant, which may be to compensate for the downregulation of SPI genes in the initial association phase of the infection.

While virulence-related genes were downregulated in the D408A mutant, general stress response mechanisms were upregulated, including phage islands, the SOS response modulators *recA* and *lexA*, and heat shock proteins (HSPs). Phage islands remain as prophages but become active when the bacterium experiences stress (42). *gtgF*, the second most highly upregulated gene in the D408A mutant, encodes the Gifsy-2 prophage DNA-damage-inducible-protein-I-like (DinI-like) protein (43). The function of prophage DinI-like proteins is still unclear (44, 45), but it has been suggested that they have a role in activation of the prophage under stress conditions (45, 46).

The SOS system is induced when damage to DNA occurs, producing single-stranded DNA. This attracts RecA, and the DNA-RecA complex facilitates expression of the SOS system by exposing the cleavage site of LexA, the main SOS system inhibitor (47). The upregulation of *recA* and *lexA* in the D408A mutant suggests DNA damage. Compounds and environmental conditions known to induce formation of single-stranded DNA include reactive oxygen species and metabolic by-products (48). These are often proteotoxic, causing proteins to unfold, which signals the overexpression of HSP (49). We speculate that the loss of function of AcrB leads to accumulation of toxic metabolites in the cytoplasm and periplasm, which results in DNA damage and protein unfolding. Consequently, this leads to upregulation of the SOS regulators and HSP. Nonetheless, it is important to note that transcription levels of the SOS effectors were not significantly changed; this suggests that the DNA damage in the D408A mutant was not sufficiently extensive to activate their transcription. Furthermore, since the total RNA samples of the wild-type strain and the D408A mutant were prepared at the same time and under the same conditions, it is unlikely that the upregulation of HSP and of DNA repair mechanisms in the mutant is an artifact of the experimental conditions. Therefore, it is most likely due to changes in the physiological state of the mutant.

We also observed upregulation of the *soxS* transcriptional regulator; this has been reported previously in an *E. coli* Δ*acrB* mutant. This was shown to be a response to intracellular accumulation of metabolites in the mutant (50). Therefore, the upregulation of *soxS* in the D408A mutant is likely to be a response to accumulation of metabolites in the bacterial cell. The accumulation of toxic metabolites in the D408A mutant is further supported by the upregulation of the *emrAB* MDR efflux pump. Similarly to AcrAB, EmrAB associates with TolC to export a variety of antibiotics and other compounds such as novobiocin, nalidixic acid, and free fatty acids, which are normally effluxed by AcrAB (51, 52). This suggests that the upregulation of *emrAB* is in response to the accumulation of toxic metabolites normally effluxed via AcrB in the D408A mutant.

We have previously reported that *ramA* and homologous RND genes *acrD* and *acrF* were significantly upregulated in an Δ*acrB* mutant (53–55). However, we did not observe upregulation of these genes in the D408A mutant. Therefore, we hypothesize that the upregulation of *ramA* and homologous RND efflux pump genes in the deletion mutant was a result of loss of the AcrB protein. The differences between the transcriptome of the D408A mutant and that of the Δ*acrB* mutant are important and lead us to propose that deletion mutants are unsuitable for studying the physiological role of membrane transporters.

We further speculate that the purpose of the downregulation of outer membrane porins in the D408A mutant is to prevent potential noxious compounds from entering the bacterium. Following this, the downregulation of tRNAs and general metabolic processes could be a defense mechanism to avoid further damage to the cells, as cells with high metabolic activity are more likely to be damaged. We propose that *Salmonella* sacrifices its virulence, a nonessential function, in order to relocate energy to essential functions such as replication, in response to the stress caused by the intracellular accumulation of toxic metabolites.

In conclusion, we present compelling evidence that the loss of efflux function of AcrB causes loss of virulence in *S*. Typhimurium. We also present evidence suggesting that the downregulation of virulence factors is an adaptation mechanism to cope with stress, which is caused by accumulation of toxic metabolites.

## MATERIALS AND METHODS

### Bacterial strains, plasmids, and construction of the *S*. Typhimurium AcrB D408A chromosomal mutant.

All *Salmonella* mutants used were derived from *S*. Typhimurium SL1344, a well-characterized representative of this serovar. Further information about the strains and plasmids can be found in Text S1 in the supplemental material. A modified version of the method described by Kim et al. (56) was used for the construction of the chromosomal mutant. Primers are listed in Table S1. The kanamycin selection marker was amplified from plasmid pT2SK, with primers MR and MF. The fragments containing the D408A mutation in AcrB were amplified from a previously constructed plasmid. The mutation cassette was assembled as previously described (56). The mutation cassette was inserted into the chromosome of *S*. Typhimurium SL1344 using pSIM18 hygromycin selection (57). Recombinants were selected by plating onto LB agar with 50 μg/ml of kanamycin. Excision of the kanamycin selection cassette from the chromosome was carried out with pACBSCE (58). Mutant candidates were screened by PCR for loss of the selection marker, and PCR amplimers were sequenced to confirm correct insertion of the mutation.

10.1128/mBio.00968-17.1 TEXT S1 Bacterial strains and plasmids used in this study. Download TEXT S1, DOCX file, 0.02 MB. Copyright © 2017 Wang-Kan et al. 2017 Wang-Kan et al. This content is distributed under the terms of the Creative Commons Attribution 4.0 International license.

10.1128/mBio.00968-17.10 TABLE S1  Primers used in this study Download TABLE S1, DOCX file, 0.01 MB. Copyright © 2017 Wang-Kan et al. 2017 Wang-Kan et al. This content is distributed under the terms of the Creative Commons Attribution 4.0 International license.

The mutant strain was sent to Microbes NG for WGS. Paired-end sequencing was carried out on the Illumina HiSeq platform. The reads were mapped to the *S*. Typhimurium SL1344 reference genome from EnsemblBacteria (assembly GCA_00210855.2) using Bowtie2. Single nucleotide polymorphism (SNP) analysis was carried out with Mpileup, and candidate SNPs were verified using Artemis (Sanger Institute, United Kingdom).

### Western blot analysis.

Protein samples from *S*. Typhimurium SL1344, its Δ*acrB* mutant, and the AcrB D408A mutant were prepared by sonication of whole cells. Protein concentration was quantified by Qubit protein assay. Ten micrograms of protein sample was used for blotting as described in reference 59.

### Growth kinetics and susceptibility of strains to AcrB substrates.

Growth kinetics were measured using a FLUOstar Optima microplate reader. The optical density at 600 nm (OD_600_) was measured every 5 min for 18 h. The MICs of AcrB substrates were determined as recommended by the European Committee on Antimicrobial Susceptibility Testing (EUCAST) (60). *Escherichia coli* ATCC 25922 was used as the control strain.

### H33342 accumulation and ethidium bromide efflux assay.

The H33342 accumulation and ethidium bromide efflux assays were carried out as previously described (61). Cultures grown to an OD_600_ of 0.6 were aliquoted into a black 96-well plate. Fluorescence was measured every minute for 100 min in a FLUOstar Optima microplate reader. Initial fluorescence of the cultures was measured for 5 min before the injection of H33342 (2.5 µM) or glucose (25 mM), respectively. For the ethidium bromide efflux assay, cultures were preloaded with 50 µg/ml of ethidium bromide and 100 μM carbonyl cyanide *m*-chlorophenylhydrazone (CCCP).

### Tissue culture.

INT-407 embryonic intestinal epithelial cells were cultured under the same conditions as in reference 62. Caco2 intestinal epithelial cells and J774 murine macrophages were cultured in Dulbecco’s modified Eagle’s medium (DMEM) with the same supplements used for INT-407 cells. Infection assays were carried out as described previously (13, 62, 63). For INT-407 and J774 cells, 2 × 10^5^ cells/ml were seeded in duplicate into 24-well plates (Costar, USA) 48 h prior to an infection assay. For assays with polarized Caco2 cells, 1.8 × 10^5^ cells/ml were polarized by growth for 14 days, with medium changed every 48 h.

### *Galleria mellonella* experiments.

A virulence comparison was performed between isolates L354 and L1804. In brief, overnight (18-h) LB broth cultures of each strain were washed in phosphate-buffered saline (PBS) before being serially diluted in PBS. CFU were determined by plating the dilutions on nutrient agar and incubating them at 37°C for 24 h. Ten *G. mellonella* larvae were infected as previously described (64). Larvae were incubated at 37°C and scored for survival (live/dead) at 0, 24, 48, 72, and 96 h postinfection. Larvae were also scored for melanization over 96 h, based on a reversed scoring of that by Tsai et al. (65), whereby a score of 4 indicated total melanization of the larvae, 2 equaled melanin spots over the larvae, 1 equaled discoloration of the tail, and a score of 0 equaled no melanization. Assays were performed in triplicate.

### Mouse experiments.

Six- to 8-week-old BALB/c mice were used to assess the virulence of the D408A mutant. Four groups of five or seven mice each were used for survival or systemic dissemination studies, respectively. *S*. Typhimurium SL1344 or its AcrB D408A mutant was inoculated orally or intraperitoneally. The oral infection was carried out with 5 × 10^7^ CFU/ml as described in reference 66, whereas the intraperitoneal inoculation was carried out with 3 × 10^3^ CFU/ml as described in reference 67. Mice were sacrificed 5 days postinfection, and spleen and liver were harvested to assess bacterial load by viable count. Survival experiments were carried out for 17 days. The research protocols, approved by the Service for Biotechnology and Animal Welfare of the Istituto Superiore di Sanità and authorized by the Italian Ministry of Health, adhered to the guidelines contained in Italian Legislative Decree 116/92, based on European Directive 86/609/EEC on Laboratory Animal Protection (decree numbers 84/12.B and 255/2012.B).

### RNA extraction, sequencing, and bioinformatics analyses.

Three biological replicates per strain were grown to an OD_600_ of 0.6 in morpholinepropanesulfonic acid (MOPS) minimal medium supplemented with 2.6 mM histidine. Total RNA was extracted using the SV total RNA isolation system (Promega, USA). The concentration of RNA and DNA in the samples was quantified by the Qubit fluorometric quantitation system. Where required, further DNase treatment was carried out using the Turbo DNA-free kit (Thermo Fisher Scientific, United Kingdom). A NanoDrop spectrophotometer and a Bioanalyzer instrument assessed purity and integrity of RNA samples, respectively. Samples were sent for RNA sequencing to the Beijing Genomics Institute (BGI; Hong Kong). Paired-end sequencing was carried out by the Illumina HiSeq 4000 system. Bioinformatics analyses were carried out exactly as described previously (68). The reference genomes were obtained from EBI (accession numbers FQ312003, HE654724, HE654725, and HE654726).

### Swimming motility assays.

Semisolid agar motility assays were carried out as described in reference 63. The inoculum was adjusted to an OD_600_ of 0.5 and stabbed into MOPS with 0.3% agar. Plates were incubated for 24 h at 37°C. The area of motility was measured with ImageJ.

### qRT-PCR and *gfp* reporter assays.

cDNA was synthesized from RNA samples, using Superscript II. cDNA was aliquoted and stored at −20°C until use. qRT-PCR was carried out with IQ SYBR green Supermix (Bio-Rad, United Kingdom) according to the instructions from the manufacturer. Differences in expression were calculated by the threshold cycle (ΔΔ*C*_*T*_) method as described previously (54). 16S rRNA and *gyrB* were used as housekeeping genes for data normalization. The *ramA-gfp* reporter plasmid was obtained from reference 53. Plasmid was inserted into the AcrB D408A mutant by electroporation. Reporter induction assays were carried out as previously described (53).

### Statistical analyses.

All experiments were repeated at least three times. Where data were represented graphically, the mean from all the experiments was plotted, except for mouse experiments, where the median was plotted. To calculate statistical significance of survival curves, a two-tailed log rank (Mantel-Cox) test was used. A two-tailed Mann-Whitney U test was used for mouse experiments, and a Student *t* test was used for all other experiments. Welch’s correction was applied to *t* tests when data sets had unequal variances. A *P* value of <0.05 was used to indicate statistical significance. This is indicated in the text as “significant.”

### Accession number(s).

The paired-end sequencing data are available in ArrayExpress (accession no. E-MTAB-5573).
