# Supplementary figures and images for: Lack of AcrB Efflux Function Confers Loss of Virulence on Salmonella enterica Serovar Typhimurium
Source: mBio. 2017 Jul 18;8(4):e00968-17. doi: 10.1128/mBio.00968-17 (PMC5516257; doi:10.1128/mBio.00968-17)

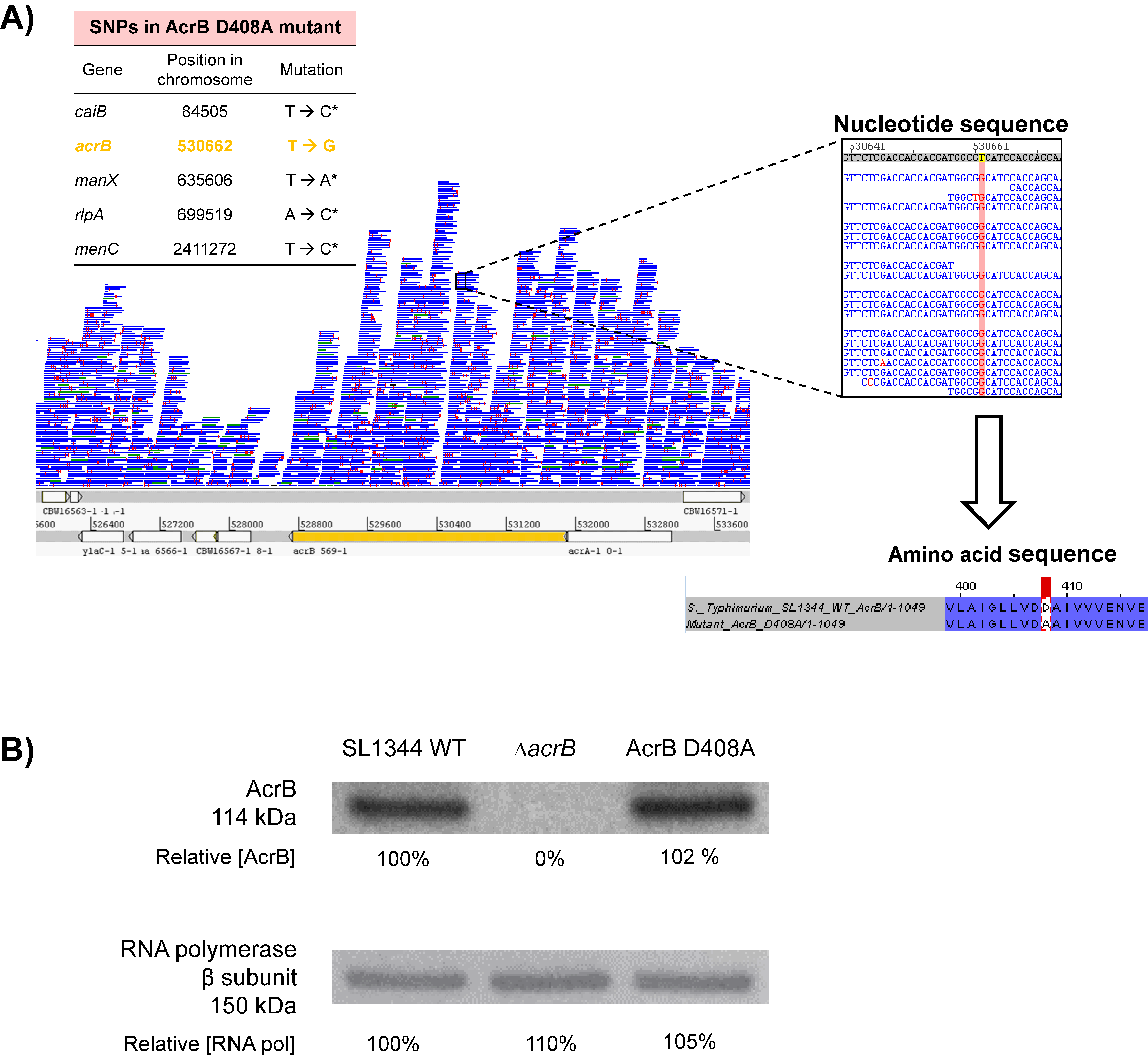

Supplement: FIG S1 [file mbo004173390sf1.tif]

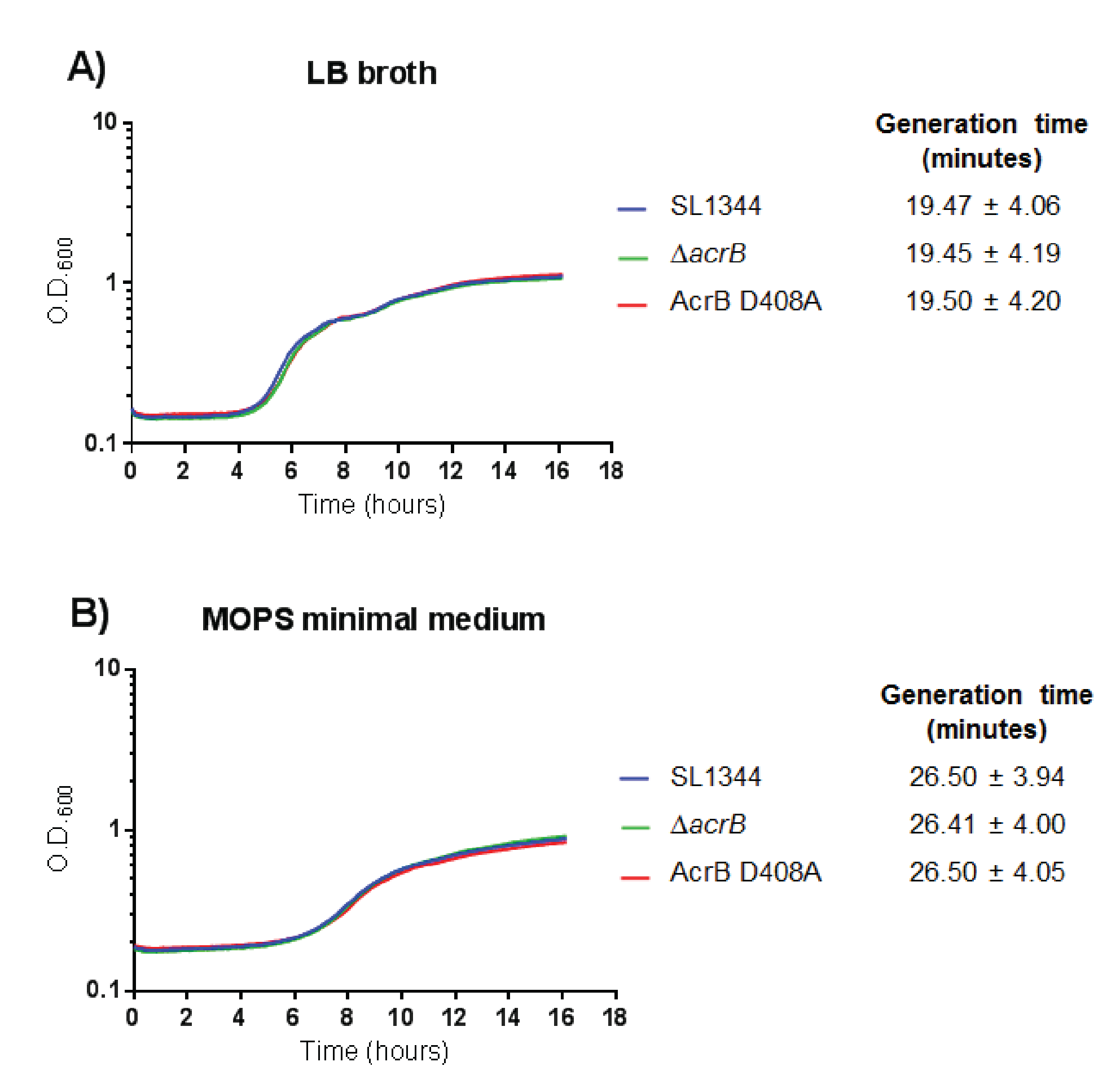

Supplement: FIG S2 [file mbo004173390sf2.tif]

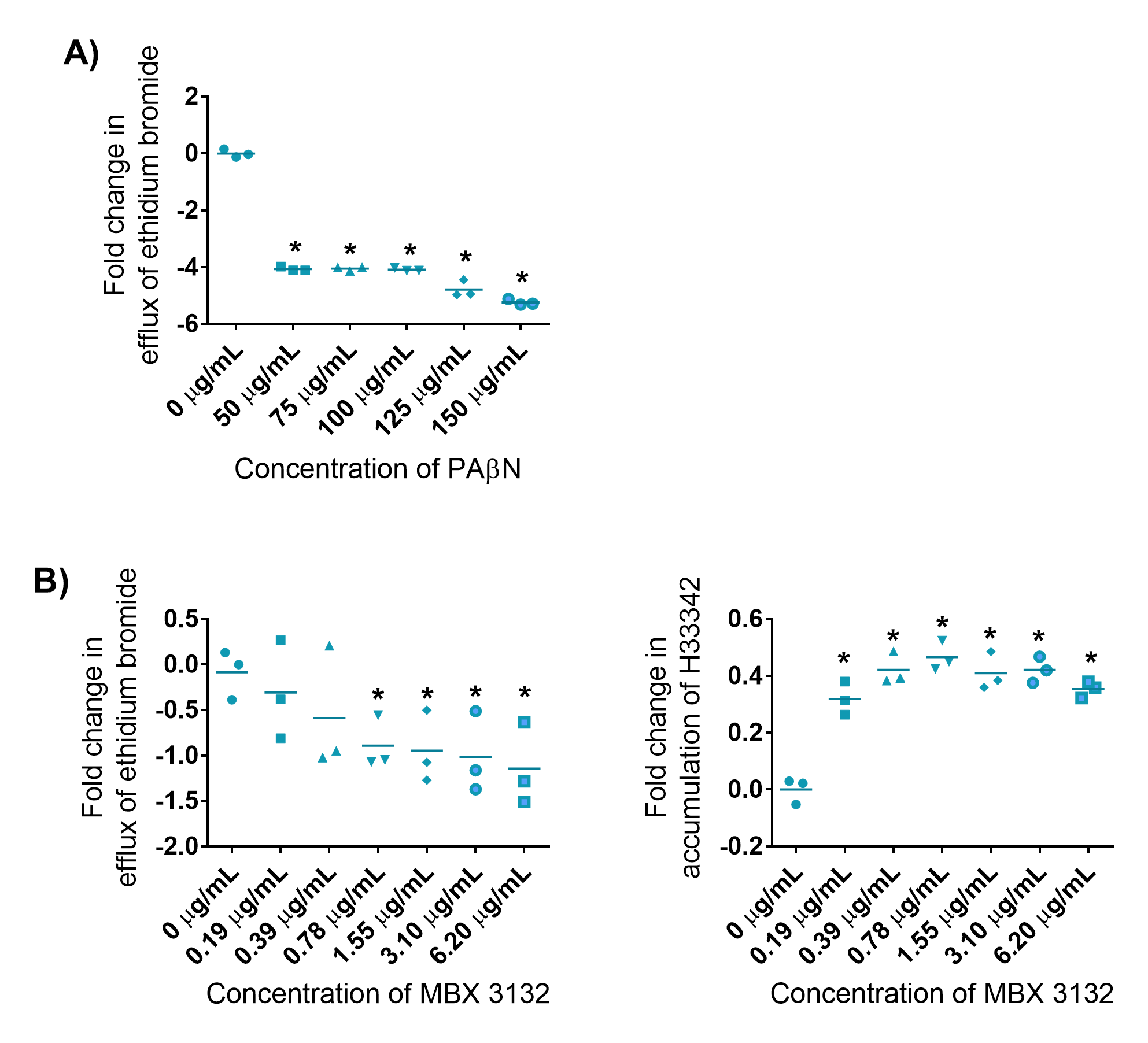

Supplement: FIG S3 [file mbo004173390sf3.tif]

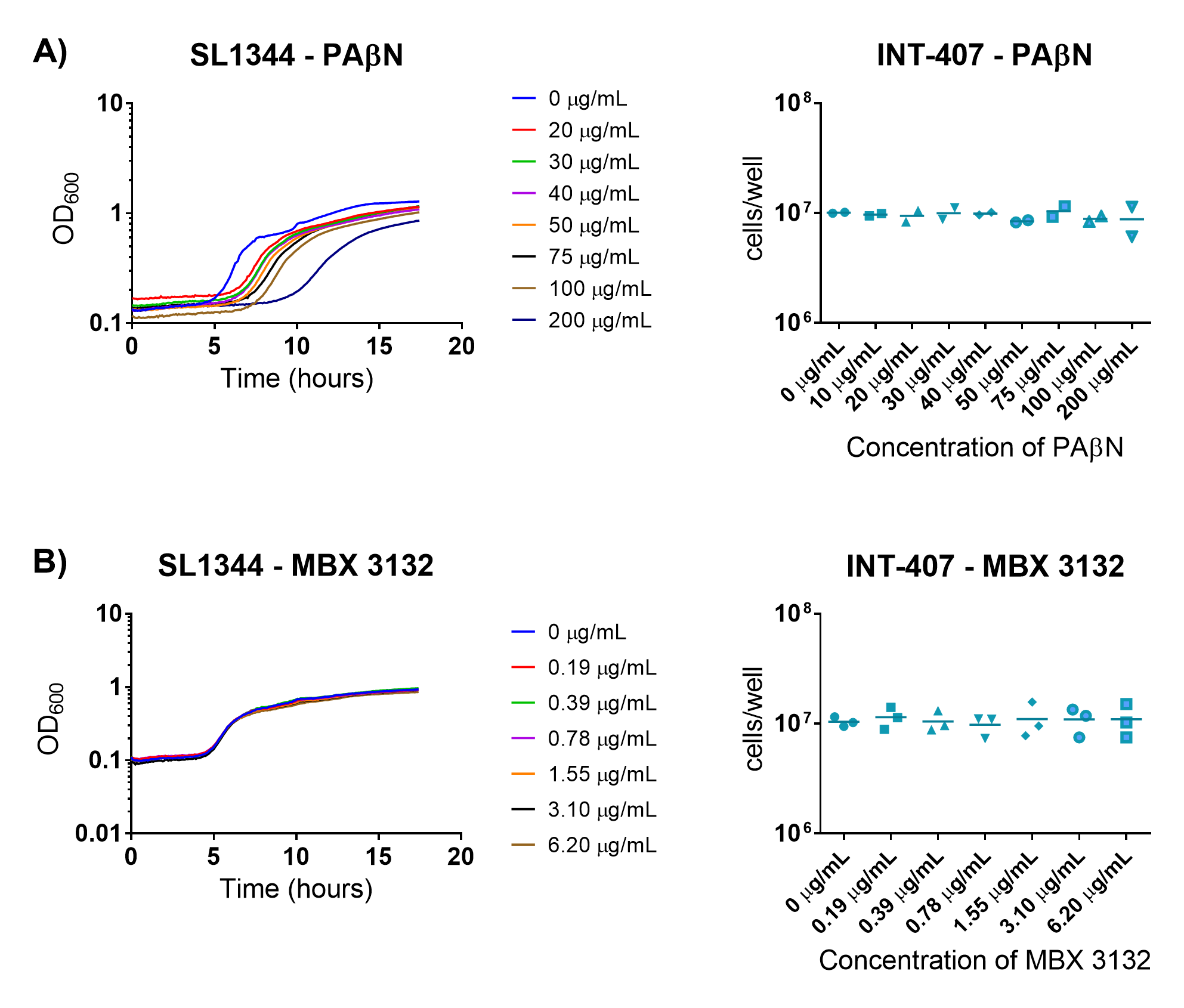

Supplement: FIG S4 [file mbo004173390sf4.tif]

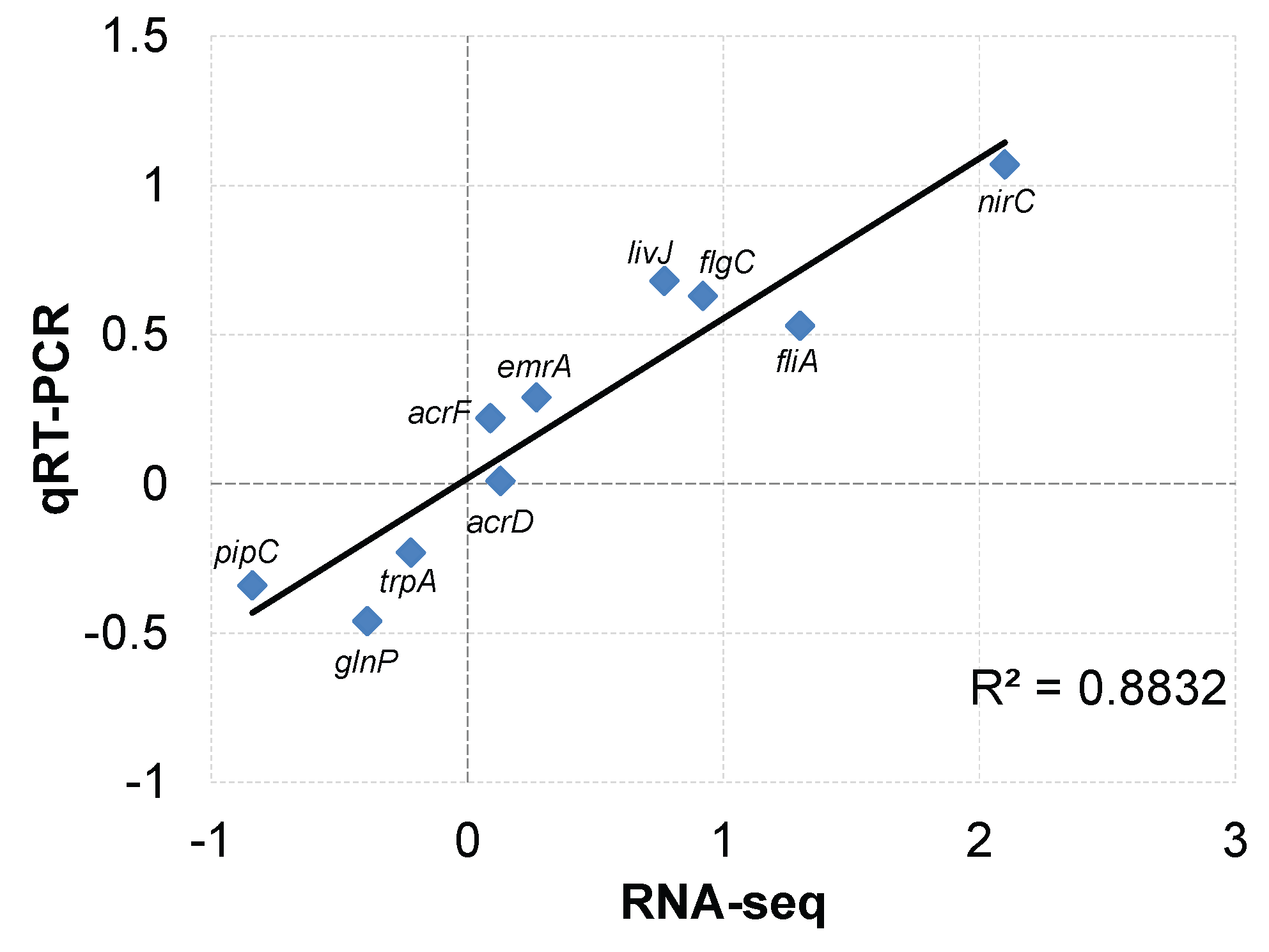

Supplement: FIG S5 [file mbo004173390sf5.tif]

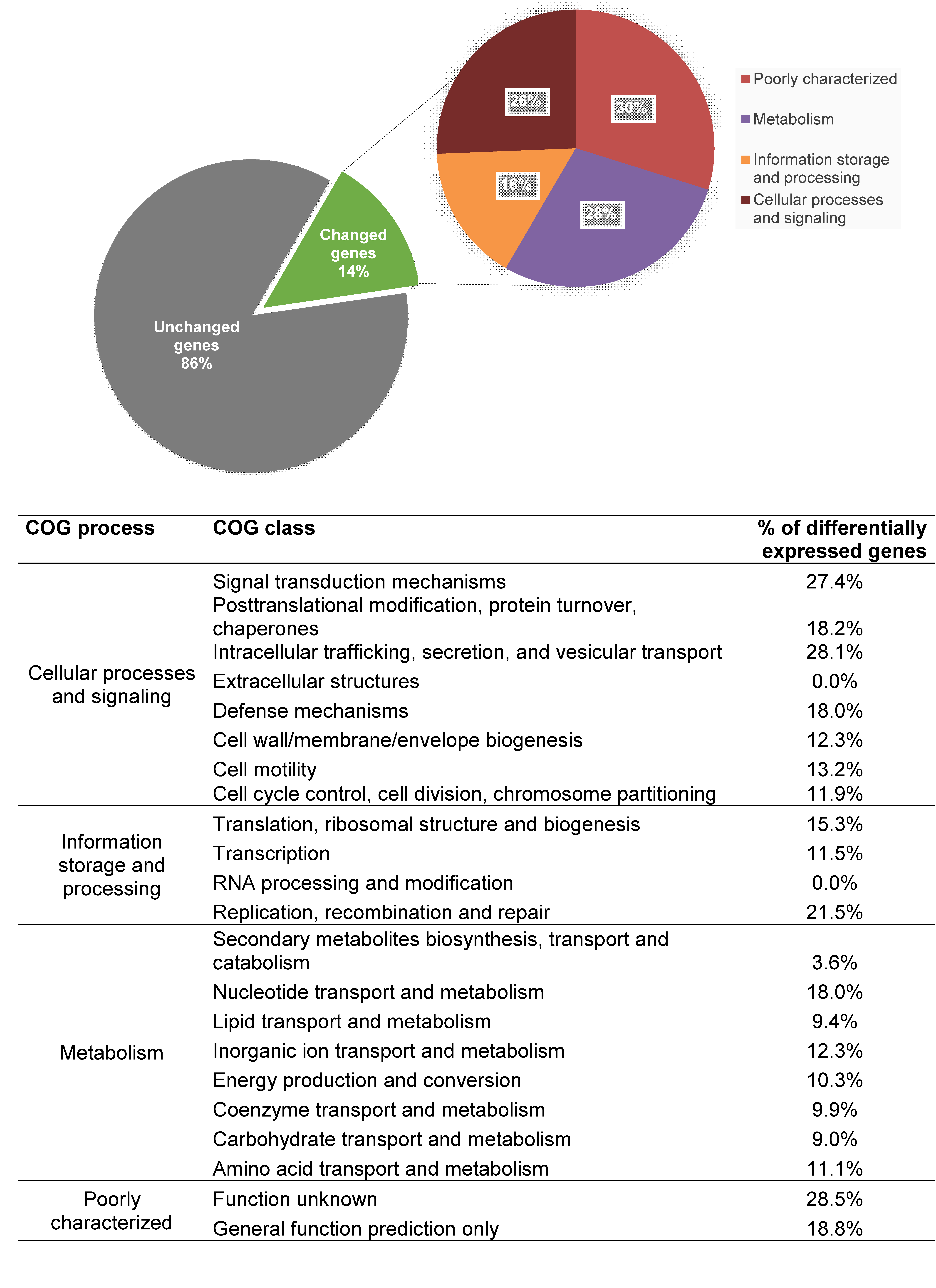

Supplement: FIG S6 [file mbo004173390sf6.tif]

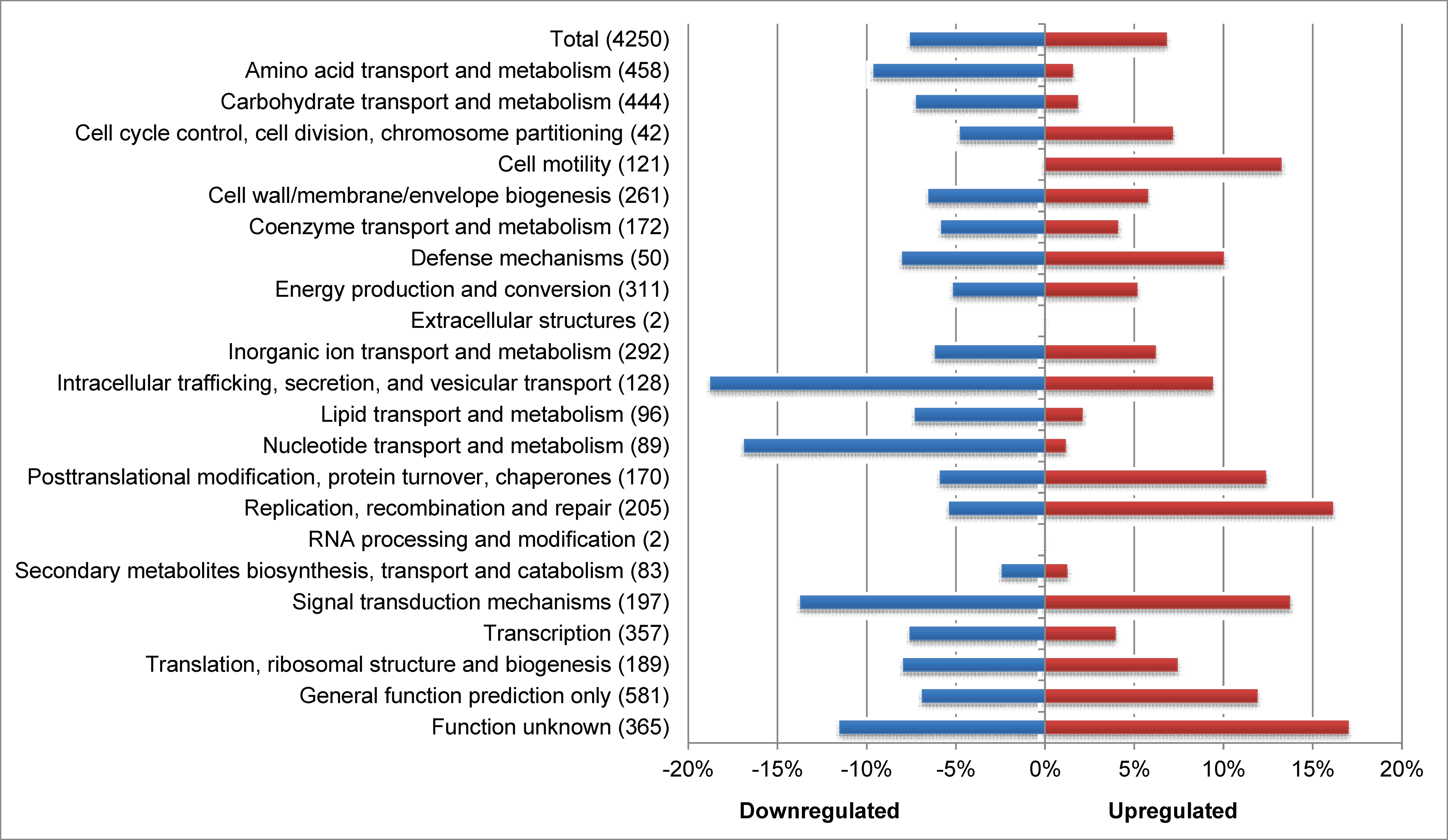

Supplement: FIG S7 [file mbo004173390sf7.tif]

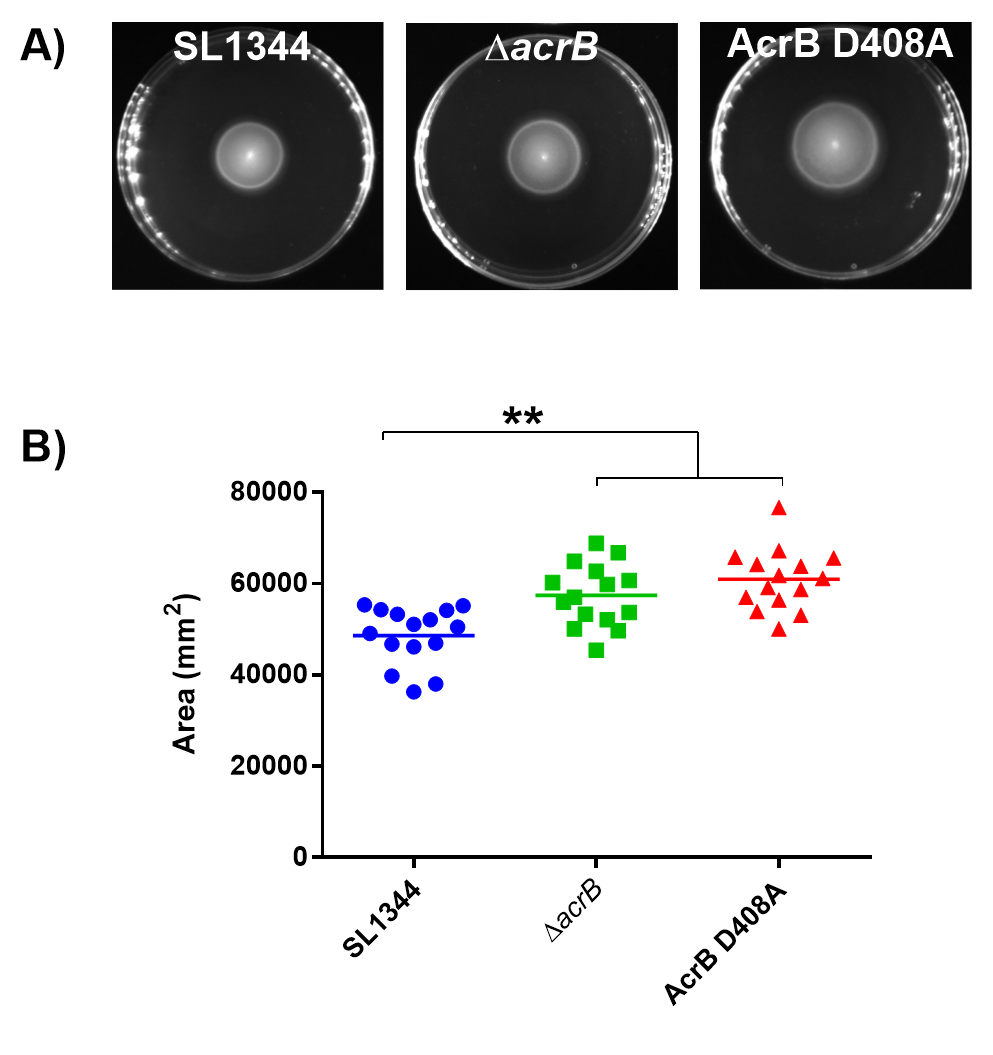

Supplement: FIG S8 [file mbo004173390sf8.tif]
