# Supplementary material for: Lack of AcrB Efflux Function Confers Loss of Virulence on Salmonella enterica Serovar Typhimurium
Source: mBio. 2017 Jul 18;8(4):e00968-17. doi: 10.1128/mBio.00968-17 (PMC5516257; doi:10.1128/mBio.00968-17)
Supplement: TEXT S1 [file mbo004173390s1.docx]

**Supplement 1 (SI1)**

**Bacterial strains and plasmids used in this study.**

The AcrB D408A mutant was constructed in *S.* Typhimurium SL1344. A previously constructed SL1344 Δ*acrB* mutant was used as phenotype control for the D408A mutant (1). *Escherichia coli* ATCC 25922 was used as a control strain for determination of antimicrobial susceptibility as recommended by EUCAST. All strains were grown in LB media at 37°C, unless specified otherwise. Plasmid pT2SK confers resistance to ampicillin and kanamycin, it contains a kanamycin resistance gene preceded by an I-SceI excision site and two translation stoppers (2). This plasmid was used for the construction of the mutation cassette.

pSim18 confers resistance to hygromycin, it also has a temperature sensitive *oriC* and encodes λ-Red recombination system under the control of *pL*; therefore, all strains harbouring this plasmid were grown at 30°C (3). This plasmid was used in insert the mutation cassette into the chromosome of SL1344. pACBSCE confers resistance to chloramphenicol and was used to excise the kanamycin resistance gene from the chromosome of the mutant. It encodes I-SceI meganuclease under the control of an arabinose-inducible promoter (4).

**References**

1. Eaves DJ, Ricci V, Piddock LJ. 2004. Expression of *acrB*, *acrF*, *acrD*, *marA*, and *soxS* in *Salmonella enterica* serovar Typhimurium: role in multiple antibiotic resistance. Antimicrob Agents Chemother 48:1145-50.

2. Kim J, Webb AM, Kershner JP, Blaskowski S, Copley SD. 2014. A versatile and highly efficient method for scarless genome editing in *Escherichia coli* and *Salmonella enterica*. BMC Biotechnol 14:1-13.

3. Lee S-C, Wang W, Liu P. 2009. Construction of Gene Targeting Vectors by Recombineering. Methods Mol Biol 530:15-27.

4. Lee DJ, Bingle LE, Heurlier K, Pallen MJ, Penn CW, Busby SJ, Hobman JL. 2009. Gene doctoring: a method for recombineering in laboratory and pathogenic *Escherichia coli* strains. BMC Microbiol 9:1-14.
