# Supplementary material for: Lack of AcrB Efflux Function Confers Loss of Virulence on Salmonella enterica Serovar Typhimurium
Source: mBio. 2017 Jul 18;8(4):e00968-17. doi: 10.1128/mBio.00968-17 (PMC5516257; doi:10.1128/mBio.00968-17)
Supplement: TABLE S1 [file mbo004173390st1.docx]

Table S1. Primers used in this study.

| **Gene/**  **Plasmid** | **Primer** | **Sequence 5’ 🡪 3’** | **Amplimer size (bp)** | **Source** |
| --- | --- | --- | --- | --- |
| pT2SK | MF | ATCTCAAGAGTGGCAGC | 1200 | Kim *et al.,* 2014 (55) |
|  | MR | TTACGCCCCGCCCTGC |  |  |
| *acrB* | D408A F | GTTGTTGGGAACCTTTGCCG | 218 | This study |
|  | D408A R | CCATCGCGATACCCACCAAT |  |  |
|  | pT2SRSM3 | GCAGGGCGGGGCGTAATCGCCATCGGCTTGCTGGTGGA | 144 | This study |
|  | SM5pT2SF | ACCGCTGCCACTCTTGAGATACGTTCTCGACCACCACGATGG | 123 | This study |
